# Investigation into Antioxidant Mechanism of *Lycium barbarum* Extract in Carbendazim-Induced PC12 Cell Injury Model through Transcriptomics and Metabolomics Analyses

**DOI:** 10.3390/foods13152384

**Published:** 2024-07-28

**Authors:** Pingxiang Liu, Ju Chen, Xing Wen, Xin Shi, Xiaoqian Yin, Jiang Yu, Yongzhong Qian, Chunlin Gou, Yanyang Xu

**Affiliations:** 1 Key Laboratory of Agro-Product Quality and Safety, Institute of Quality Standards and Testing Technology for Agro-Products, Chinese Academy of Agricultural Sciences, Beijing 100081, China; liupingxiangtri@163.com (P.L.); 18220528265@163.com (J.C.); 15029240387@163.com (X.W.); 17860269372@163.com (X.Y.); qianyongzhong@caas.cn (Y.Q.); 2 Institute of Quality Standard and Testing Technology for Agro-Products, Shandong Provincial Key Laboratory of Test Technology on Food Quality and Safety, Shandong Academy of Agricultural Sciences, Jinan 250100, China; 3 Faculty of Printing and Packaging and Digital Media, Xi’an University of Technology, Xi’an 710048, China; yupp126@163.com; 4 Institute of Quality Standard and Testing Technology for Agro-Products of NingXia, Yinchuan 750002, China; sxdewp@163.com

**Keywords:** *Lycium barbarum* L., antioxidant activity, CBZ-induced injury, transcriptomics, metabolomics

## Abstract

*Lycium barbarum* L., an important functional food in China, has antioxidant and antiaging activity. However, the exact antioxidant activity mechanism of *Lycium barbarum* extracts (LBE) is not well understood. Therefore, a carbendazim (CBZ)-induced PC12 cell injury model was constructed and vitrificated to study the antioxidant activity of fresh LBE on the basis of extraction parameter optimization via the full factorial design of experiments (DOE) method. The results showed that the pretreatment of PC12 cells with LBE could reduce the reactive oxygen species (ROS) level by 14.6% and inhibited the mitochondrial membrane potential (MMP) decline by 12.0%. Furthermore, the integrated analysis revealed that LBE played an antioxidant role by activating oxidative phosphorylation (OXPHOS) and restoring MMP, maintaining the tricarboxylic acid (TCA) cycle stability, and regulating the GSH metabolic pathway. The results of the present study provide new ideas for the understanding of the antioxidant function of LBE from a global perspective.

## 1. Introduction

*Lycium barbarum* L. (LB), also known as Chinese boxthorn, goji berry, or barbary wolfberry, is a plant that is indigenous to the arid and semi-arid regions of Northwestern China [1,2]. The fruit of LB, which is an orange-red berry and has a delightful sweet and tangy taste, has long been used as a medicine and functional food in China [1]. In a previous study, 42 different fruit samples were studied, and the LB fruit produced in Zhongning, Ningxia, China was found to have good quality and higher content of LB polysaccharides (LBPs) and most micronutrients, along with an appropriate sugar-to-alkali ratio [3]. Recently, the LB fruit has received increasing attention due to its remarkable pharmacological properties, including its antioxidant, antiglycemic, neuroprotective, hepatoprotective, and immunomodulatory activity [4]. The primary contributors to the significant antioxidant activity of LB are carotenoids, flavonoids, ascorbic acid and its derivatives, and polyphenols [5]. Among these compounds, 2-O-β-D-glucopyranosyl-L-ascorbic acid (AA-2βG) and chlorogenic acid (CGA) serve as important functional components of LB. AA-2βG, the characteristic chemical constituent of LB, is a novel and stable derivative of vitamin C that was first isolated and purified from dried LB fruit by Toyoda-Ono et al. [6]. LB fruit is typically dried before it is stored or sold. However, this drying process can deplete the functional components of LB fruit but is expected to emerge as the preferred choice [7]. Unfortunately, LB is susceptible to diseases such as root rot, anthracnose, and powdery mildew. CBZ is a broad-spectrum fungicide that effectively controls fungal diseases in a wide range of crops [8]. Notably, CBZ is commonly utilized during the cultivation of LB to ensure its yield and quality. However, the widespread use of CBZ can leave drug residues in LB fruits, posing potential health risks to consumers. It is necessary to comprehensively assess the antioxidant activity of LB in the presence of CBZ residues.

Currently, classical chemical methods such as the 2,2-diphenyl-1-picrylhydrazyl (DPPH) free radical scavenging, 2,2′-azino-bis(3-ethylbenzothiazoline-6-sulfonic acid) (ABTS) free radical scavenging, ferric reducing antioxidant power (FRAP), and oxygen radical absorbance capacity (ORAC) assays are the most commonly employed approaches for the evaluation of the antioxidant capacity of natural products [9]. However, these chemical methods cannot simulate the actual physical environment and fail to elucidate the relationship between the antioxidant and pharmacological effects of natural products. Interestingly, a cell culture-based activity screening model has shown exceptional value in examining the antioxidative potential of natural substances. Specifically, this evaluation method can reveal the intracellular mechanism of oxygen free radical scavenging by evaluating the entry of substances across the cell membrane, their absorption behavior, and their interactions with biomacromolecules such as carriers or enzymes. Hence, this method can provide an accurate evaluation of the antioxidant activity of target compounds and effectively explain the mechanism of action [10]. Free radicals have been linked to aging-related central neurodegenerative diseases, such as Alzheimer’s disease and Parkinson’s disease [11]. PC12 cells, a line of rat pheochromocytoma cells, can grow and differentiate into neurite-bearing cells that have the basic biological characteristics of neurons. Thus, they are an ideal cellular model for the evaluation of antioxidant activity.

Furthermore, cellular evaluation methods have recently been combined with multi-omics techniques, such as transcriptomics, metabolomics, lipidomics, and proteomics, and these have recently been employed to investigate antioxidant mechanisms in cellular models [12]. Among these techniques, transcriptomics is a tool for genome analysis that utilizes microarray chips and sequencing technology to quantitatively analyze the total RNA present in cells. Transcriptomics analysis can comprehensively and rapidly provide the sequence information of intracellular RNA in different states. Its high-throughput nature, exceptional sensitivity, and versatile functionality have led to its widespread application in scientific research, clinical diagnosis, and pharmaceutical development [13]. Meanwhile, metabolomics can be used to comprehensively analyze the metabolite composition, serving as a valuable tool for biomarker identification and the elucidation of drug mechanisms. Previous studies have demonstrated that metabolomics can be used to elucidate the mechanisms underlying the effects of traditional Chinese medicines such as Zhishi-Xiebai-Guizhi decoctions [14] and luteolin [15] in ameliorating intracellular oxidative stress injury. Therefore, a cell culture-based activity screening model combined with multi-omics techniques could offer more reliable results and enable a deeper understanding of the antioxidant capacity mechanisms of natural products.

Thus, in this study, a CBZ-induced PC12 cell injury model was constructed and validated to study the protective effect of LBE. Additionally, the mechanism of its antioxidant activity was investigated using a combination of molecular biology, transcriptomics, and metabolomics.

## 2. Materials and Methods

### 2.1. Materials and Chemicals

RPMI-1640 medium, fetal bovine serum (FBS), 0.25% trypsin, streptomycin sulfate, and penicillin were purchased from Gibco (Thermo Fisher Scientific, Waltham, MA, USA). CBZ, AA-2βG, and CGA standards with purity of 99.6%, 99.5%, and 98.9%, respectively, were purchased from Alta Scientific (Tianjin, China). The Cell Counting Kit-8 (CCK8) was purchased from Dojindo (Kumamoto, Japan). The CellROX^®^ Oxidative Stress Reagent Kits and TRIzol Reagent were purchased from Thermo Fisher (Waltham, MA, USA). Methanol and dimethyl sulfoxide (DMSO) were purchased from Sigma-Aldrich (St. Louis, MO, USA). The enhanced mitochondrial membrane potential (MMP) assay kit with JC-1 and Hoechst 33342 Staining Solution for Live Cells were purchased from Beyotime (Shanghai, China).

### 2.2. Cell Culture

The PC12 rat adrenal pheochromocytoma cell line was obtained from the Cell Bank of Type Culture Collection of the Chinese Academy of Science (Shanghai, China). The PC12 cells were cultivated in RPMI-1640 medium supplemented with 10% FBS and 1% antibiotics, which included 100 U/mL penicillin and 100 μg/mL streptomycin. The incubation of the PC12 cells was carried out at a temperature of 37 °C under conditions of 95% relative humidity and 5% CO_2_.

### 2.3. Construction and Verification of CBZ-Induced PC12 Cell Injury Model

A total of 10.6 mg of CBZ standard substance was added to a mixture of 4 mL of methanol and 6 mL of DMSO to obtain the CBZ stock solution. PC12 cells were plated at a density of 1 × 10^5^ cells/mL in a 96-well plate. Following an incubation period of 24 h, the cells were exposed to various concentrations (0.3, 1, 3, 10, 30, 100, and 300 μM) of CBZ for 24 h, and the cell viability was subsequently measured. A total of 10.38 mg of AA-2βG standard substance was added to a mixture of 4 mL of methanol and 6 mL of DMSO to obtain the AA-2βG standard solution. This solution was diluted with complete culture medium to obtain corresponding concentration gradients (180, 120, 60, and 30 μM). Similarly, 10.4 mg of CGA standard substance was added to a mixture of 4 mL of methanol and 6 mL of DMSO to obtain the CGA standard solution. The corresponding solution was diluted with complete culture medium to obtain the desired concentration gradients (180, 120, 60, and 30 μM) of CGA solution. The control group consisted of cells that were treated with 0.1% DMSO.

CGA and AA-2βG, two typical functional components in LB, were selected to study their protective effects on oxidative stress-damaged cells. The cells were categorized into four groups: blank, control, CBZ-induced, and treatment. The control and CBZ-induced groups were added to 100 μL of complete culture medium. Meanwhile, the treatment group received a 2 h pretreatment with varying concentrations of CGA and AA-2βG. Following this, both the CBZ-induced group and the treatment group were exposed to 100 μL of CBZ for a duration of 24 h. Subsequently, the cellular viability was assessed.

### 2.4. Preparation of LBE Based on Full Factorial Experimental Design

#### 2.4.1. LB Materials

The fresh LB fruit was harvested in Ningxia and transported in a temperature-controlled environment to the laboratory. The fresh LB fruit was briefly rinsed with purified water and dried with paper. Then, the samples were pre-cooled in liquid nitrogen and then transferred to a freezer at −80 °C, where they were pre-frozen overnight. Subsequently, they were processed in a vacuum freeze-dryer for 4 days. The resulting freeze-dried samples were pulverized using a mortar and pestle, sieved through a 20-mesh screen, and stored at −80 °C until analysis.

#### 2.4.2. Preparation of LBE

Exactly 100 mg LB powder was weighed and mixed with 1 mL complete culture medium. The mixture was thoroughly vortexed and subjected to sonication for 1 min in an ultrasonic water bath (Shumei, Kunshan, China). During the extraction process, three experimental factors were chosen, namely the sonication time (t), sonication temperature (T), and sonication intensity (E), and two levels were set for each factor as follows: t (15, 30 min), T (30, 50 °C), and E (60, 100 W). The ranges of the variables were selected based on a previous study [16]. The DOE trial design is shown in Table S1. After being subjected to 8 different pretreatment conditions, the sample solutions were centrifuged at 10,000× *g* rpm for 10 min at room temperature in a centrifuge (Sigma, Rödermark, Germany). Subsequently, the supernatant was then transferred into 1.5 mL EP tubes and kept at a temperature of 4 °C. The FRAP assay and DPPH free radical scavenging activity assay were used to assess the antioxidant activity.

The antioxidant activity of LBE was determined by an antioxidant capacity assay kit using the FRAP method (Beyotime Institute of Biotechnology, Shanghai, China). We employed a method similar to that in reference [17]. First, 5 μL LBE solution (2000 μg/mL) was added to 180 μL FRAP reagent. The optical density (OD) values were measured at 593 nm using a microplate reader (TECAN, Männedorf, Switzerland) after incubation at 37 °C for 5 min. The concentration of the antioxidants with an iron-reducing capacity was expressed as the final result, calculated based on the standard curve for FeSO_4_-7 H_2_O at concentrations of 0.3, 0.6, 0.9, 1.2, and 1.5 mmol/L, equivalent to 1.0 mmol/L FeSO_4_. Each sample was prepared in triplicate.

The DPPH free radical scavenging capacity was measured as already described [18]. A 400 μL LBE aliquot (2000 μg/mL) was mixed with 400 μL of DPPH–ethanol solution. The mixtures were left to stand for 30 min at room temperature in a dark room, followed by centrifugation at 10,000× *g* rpm for 5 min. A 200 μL aliquot was then transferred to a 96-well plate, and the absorbance was taken at 517 nm (*A_sample_*). The control group and blank group were mixed with methanol and the aliquot (*A_control_*) or methanol and the DPPH reagent (*A_blank_*), respectively, in the same volume. The standard curve was drawn using various concentrations of Trolox standard solutions. The DPPH radical scavenging assay was performed by comparing the sample to the control, using the following Formula (1):

(1) $$\mathrm{DPPH}\;\mathrm{radical}\;\mathrm{scavenging}\;\mathrm{assay}\;(\%)=\left(1-\frac{A_{sample}-A_{control}}{A_{blank}}\right)\times 100\%$$

Based on the Minitab statistical software ( v21.4.2), an analysis was performed on the data of the DPPH radical scavenging rates from the eight different pretreatment conditions of LBE to determine the significant influencing factors affecting the extraction efficiency of fresh LB.

#### 2.4.3. Detection of Total Phenolic and Flavonoid Content in LBE

The total phenolic content was determined according to the Folin-Ciocalteu colorimetric method, and flavonoids were analyzed using the NaNO_2_-Al(NO_3_)_3_-NaOH chromo-genic method. The extract solution of LB was obtained by ultrasound at 30 °C and 100 W for 15 min. The total phenolic and total flavonoid content was expressed as milligram gallic acid equivalents per gram of LB (mg GAE/g LB) and milligram rutin per gram of LB (mg rutin/g LB), respectively. The specific operations were in line with the Micro Plant Total Phenol Assay Kit and Micro Plant Flavonoids Assay Kit (Solarbio, Beijing, China).

### 2.5. Optimization of LBE Dose

Four groups, namely the blank group, control group, CBZ-induced group, and LBE treatment group, were used in the present study. The use of the blank group, control group, and CBZ-induced group was in accordance with Section 2.3. The treatment group was preincubated with varying concentrations of LBE (100, 500, 1000, 1500, and 2000 μg/mL) for 2 h. Then, CBZ was added to the wells of both the CBZ-induced group and the treatment group. After a 24 h incubation period, the cellular viability was assessed.

### 2.6. Cell Viability Assay

The CCK-8 assay was utilized to assess cell viability. After the exposure period, a volume of 10 μL CCK-8 solution was introduced into each well of the 96-well plates. After incubation for 2 h, the OD of each well was determined at 450 nm. The cell viability was calculated with the following Formula (2), where the treatment group consisted of cells treated with CBZ or LBE + CBZ, the control group consisted of cells treated with the culture medium, and the blank group consisted of only the culture medium:

(2) $$cell\;viability\left(\%\right)=\frac{\left(OD\;of\;treatment\;group-OD\;of\;blank\;group\right)}{\left(OD\;of\;control\;group-OD\;of\;blank\;group\right)}\times 100\%$$

### 2.7. ROS Level Assays

Reactive oxygen species (ROS) play essential roles in various biological processes, such as cell signaling and the immune response. However, cellular oxidative stress occurs when excessive oxygen radicals are generated. When the ROS concentrations are not regulated by internal defense mechanisms, oxidative injury will occur [19]. The intracellular ROS levels were measured using a cell-based high-content screening assay. PC12 cells were seeded in black 96-well plates. The treatment group received a 2 h pretreatment with 1500 μg/mL LBE, which was obtained as described in Section 2.5. Meanwhile, for the control and CBZ-induced groups, 100 μL of complete culture medium was added. Following this, both the CBZ-induced group and the treatment group were exposed to CBZ (15.9 μmol/L) for a duration of 24 h.

The 96-well plates were incubated for 2 h after staining with CellROX Green and Hoechst 33342. Then, the cells were washed with PBS after removing the dyes. Finally, 100 μL PBS was added to each well for a high-content test. The ROS test’s excitation wavelength was 460-490 nm, and the emission wavelength was 500-550 nm. The results are expressed as the average fluorescence intensity of each cell.

### 2.8. Detection of MMP

When the mitochondrial membrane potential (MMP) is relatively elevated, JC-1 forms a polymer in the mitochondrial matrix, resulting in the emission of red fluorescence. Conversely, when the MMP decreases, JC-1 is unable to aggregate within the matrix, resulting in green fluorescence production [20]. Hence, the proportion of red and green fluorescence is frequently employed to assess alterations in MMP. The procedures for cell treatment were identical to those described in Section 2.7. Following the completion of the treatment, cells were harvested and suspended in culture media at a concentration of 1 × 10^5^ cells/mL before being subjected to incubation with JC-1 staining solution (5 μg/mL) at 37 °C for a duration of 20 min. After applying staining, the FACS flow cytometry technique was employed to analyze the intensity of the red fluorescence emitted by the JC-1 aggregates. This was accomplished by utilizing an excitation wavelength of 525 nm and an emission wavelength of 590 nm. Additionally, the study employed a 490 nm excitation wavelength and a 530 nm emission wavelength to measure the green intensity of JC-1 monomers. The FlowJo software version 9.3.2 (TOMY Digital Biology, Tokyo, Japan) was utilized to calculate the relative ratio between the red and green fluorescent intensities, enabling us to determine the extent of mitochondrial depolarization.

### 2.9. Transcriptome Sequencing and Analysis

Transcriptomics cell samples were cultured in 25 cm^2^ cell culture flasks (2.5 × 10^5^ cells/mL) for 24 h. The subsequent cell treatment methods were the same as in Section 2.7. After 26 h of treatment, the transcriptomics samples were collected. The RNA Nano 6000 Assay Kit from the Bioanalyzer 2100 system (Agilent Technologies, Santa Clara, CA, USA) was used to evaluate the RNA integrity. The library was constructed according to the instructions of Illumina’s NEBNext^®^ UltraTM RNA Library Prep Kit, and then the library construction and quality control of the PC12 cell samples were carried out using ordinary NEB library construction. The TruSeq PE Cluster Kit v3-cBot-HS was implemented on a cBot Cluster Generation System (Illumia, San Diego, CA, USA) to cluster the index-coded samples. After clustering, 150 bp paired-end reads were generated by sequencing the library preparations on an Illumina Novaseq platform.

### 2.10. High-Throughput Targeted Metabolomics Analysis

The performance of the metabolomics analysis of the samples was similar to the procedure described in Section 2.9. High-throughput targeted metabolomics and multiple reaction monitoring (MRM) analysis on 2251 compounds were performed with UPLC-MS/MS. Cell samples were harvested by centrifugation at 1500× *g* rpm for 5 min at 4 °C, following which the liquid portion above was separated and discarded. To the remaining sample, 1.2 mL of ice-cold acetonitrile–methanol–H_2_O (2:2:1, containing isotope internal standards) was added and the tubes placed on dry ice to pre-cool. The samples were homogenized at 40 Hz for 4 min, followed by sonication in an ice water bath for 5 min. This process was repeated three times with alternating cycles of homogenization and sonication. The samples were subjected to sonication in an ice water bath for 15 min, followed by incubation at −40 °C for 2 h. The samples then underwent centrifugation at 12,000× *g* rpm and 4 °C for 15 min. Subsequently, 1000 μL supernatant from each sample was transferred to a new tube and dried by centrifugation. To reconstitute the dried samples, 110 μL of 60% acetonitrile was added to the tube, followed by centrifugation at 12,000× *g* rpm for 15 min at a temperature of 4 °C. Finally, the above supernatant was moved into a glass receptacle to undergo UPLC-MS/MS analysis. Mixtures of standard metabolites were prepared as QC samples. Subsequently, a total of six standard calibration solutions were obtained through the gradual dilution of the above mixed standard solution, which contained an isotopically labeled internal standard mixture at the same concentration as the samples. The standard curves of 251 compounds are shown in Table S2.

The separation was performed on a Waters Atlantis Premier BEH Z-HILIC column (1.7 µm, 2.1 mm × 150 mm) using an UPLC system (PREMIER, Waters). The composition of mobile phase A consisted of a solution containing 10 mmol/L ammonium acetate in a mixture of H_2_O and acetonitrile (8:2), while mobile phase B was a solution containing 10 mmol/L ammonium acetate in a mixture of H_2_O and acetonitrile (1:9). The pH was adjusted to 9 with ammonium hydroxide. The autosampler temperature was kept at 40 °C, while the autosampler temperature was then set at 8 °C. An AB Sciex QTrap 6500+ mass spectrometer was applied to monitor the MRM information of the metabolites. The ion source parameters were as follows: ion spray voltage: +5000 V/−4500 V, temperature: 500 °C, curtain gas: 35 psi, ion source gas 1: 50 psi, ion source gas 2: 50 psi.

### 2.11. Statistical Analysis

All results are represented as the mean ± RSD. The bar charts and dose–response curves were drawn using Excel 2019 and Origin 2019b, respectively. The final dataset for targeted metabolomics was imported into the SIMCA16.0.2 software for multivariate analysis. The database of KEGG (http://www.genome.jp/kegg/, 4 April 2023) was used for pathway enrichment.

## 3. Results and Discussion

### 3.1. Construction of the Oxidative Stress Injury PC12 Cell Model

#### 3.1.1. Construction of the CBZ-Induced PC12 Cell Injury Model

**Figure 1:**
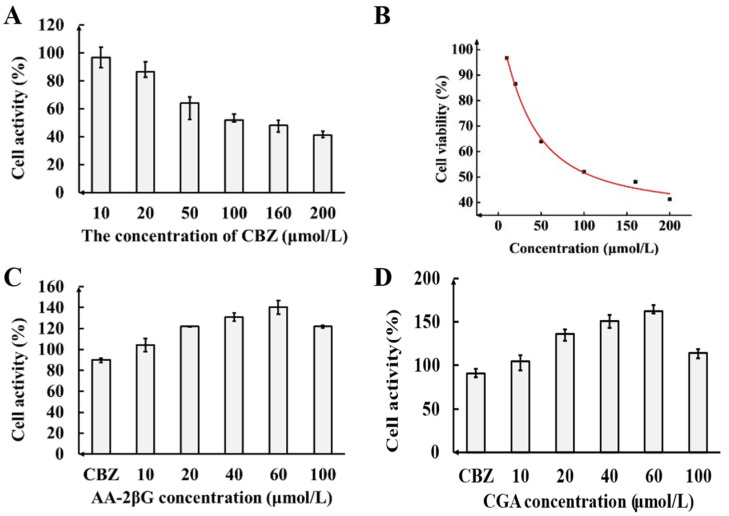
Effects of different concentrations of CBZ on activity of PC12 cells (**A**) and dose–response curve of PC12 cells exposed to CBZ alone for 24 h (**B**); effects of different concentrations of AA-2βG (**C**) and CGA (**D**) on activity of PC12-damaged cells.

The viability of PC12 cells after CBZ treatment is shown in Figure 1A,B. The R^2^ value of the “concentration–cell viability” curve in Figure 1B is 0.995, indicating the effectiveness and reliability of the model. The findings show that CBZ, within the concentration range of 10–200 μmol/L, significantly reduced the cell viability in a dose-dependent manner. Previous studies have reported low residual levels of CBZ in LB, ranging from 0.0008 to 1.927 mg/kg [8]. Notably, the concentration–cell activity curve revealed that the IC_10_ for CBZ was 15.9 μmol/L (3.040 mg/L), showing a minimal difference from the residue level in LB. Therefore, a concentration of 15.9 μmol/L was selected for the induction of the cell injury model in PC12 cells by CBZ.

#### 3.1.2. Verification of the Oxidative Stress Model in PC12 Cells

As shown in Figure 1, the viability of PC12 cells after treatment with CBZ decreased by 10% compared with that in the control group. Compared to the CBZ group, pretreatment with AA-2βG and CGA (10–100 μmol/L) for 2 h significantly improved the viability of CBZ-treated PC12 cells. Notably, the 60 μmol/L AA-2βG + 15.9 μmol/L CBZ group exhibited the highest viability (140%, Figure 1C), similar to that in the 60 μmol/L CGA + 15.9 μmol/L CBZ group (162%, Figure 1D). These findings indicate that the pretreatment with AA-2βG and CGA effectively suppressed the cell damage induced by CBZ in PC12 cells. In addition, reduced cell viability was observed at AA-2βG and CGA concentrations of 100 μmol/L. This may have been because the cytotoxicity was concentration-dependent, and higher concentrations (100 μmol/L) of AA-2βG and CGA were toxic to PC12 cells damaged by oxidative stress. These results indicate that the PC12 oxidative stress cell model induced by CBZ is reliable for the investigation of the antioxidant effects of fresh LB.

### 3.2. Optimization of Fresh LBE Preparation Conditions

#### 3.2.1. Optimization Based on FRAP and DPPH Assays

**Figure 2:**
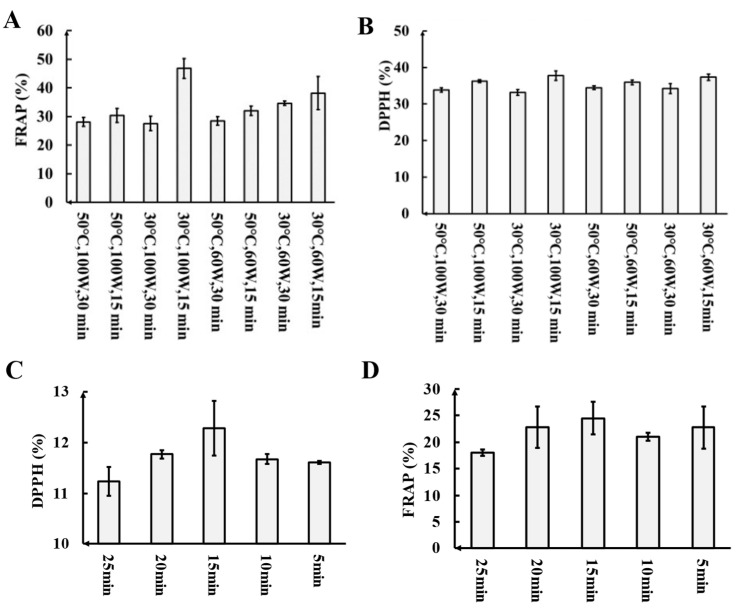
Comparison of FRAP (**A**) and DPPH scavenging activity (**B**) with 2 mg/mL LBE; comparison of DPPH scavenging activity (**C**) and FRAP (**D**) of LBE after different extraction times.

The overall antioxidant capacity of fresh LBE obtained using eight different extraction conditions was determined by the FRAP method. As shown in Figure 2A, the highest total antioxidant capacity was observed after fresh LBE treatment for 15 min at 30 °C with 100 W of ultrasound, resulting in a FRAP value of 49.48%.

The DPPH free radical scavenging rates of fresh LBE obtained using eight different extraction conditions were also evaluated, and the relative standard deviation (RSD) was calculated. As shown in Figure 2B, all LBE samples produced with the eight types of ultrasonic extraction conditions exhibited DPPH scavenging activity. Notably, the extracts obtained using the conditions of 15 min, 30 °C, and 100 W ultrasound displayed the highest DPPH free radical scavenging activity (11.65%), consistent with the FRAP results.

#### 3.2.2. Optimization of the Extraction Parameters Based on the Full Factorial DOE

The rates of DPPH free radical scavenging shown by the fresh LBE solutions produced using the eight different pretreatment conditions were analyzed with the Minitab software (v21.4.2), revealing that the standardized Pareto effect of the ultrasonication time was higher than the reference line at 2.776, indicating statistical significance with the greatest effect on the extraction efficiency. To further verify the effect of the ultrasonication time on the extraction efficiency, a single-factor test was conducted to optimize the duration of ultrasonication. During this evaluation, the temperature and intensity of the ultrasonication were maintained at 30 °C and 100 W, respectively, while extraction times of 5, 10, 15, 20, and 25 min were used. Based on the outcomes of the DPPH and FRAP experiments (Figure 2C,D), an ultrasonication time of 15 min was found to be optimal. Therefore, the optimal extraction conditions for fresh LBE were an ultrasonication duration of 15 min at a temperature of 30 °C and an intensity of 100 W.

#### 3.2.3. Total Phenolic and Flavonoid Content of LBE

The LB extracts were obtained using 100 W ultrasonication at 30 °C and 100 W for 15 min. The standard curves for the total phenols and flavonoids are shown in Figure S1. The total phenolic content was 0.4298 ± 0.004 (gallic acid equivalent) mg/g LB and the total flavonoid content was 0.5102 ± 0.002 (rutin equivalent) mg/g LB. The content of total phenols and flavonoids was consistent with the reports of Prodromos [21] and Ma [22]. This proves that the extraction method based on the full factorial DOE is feasible.

### 3.3. Protective Effects of LBE on CBZ-Damaged PC12 Cells

**Figure 3:**
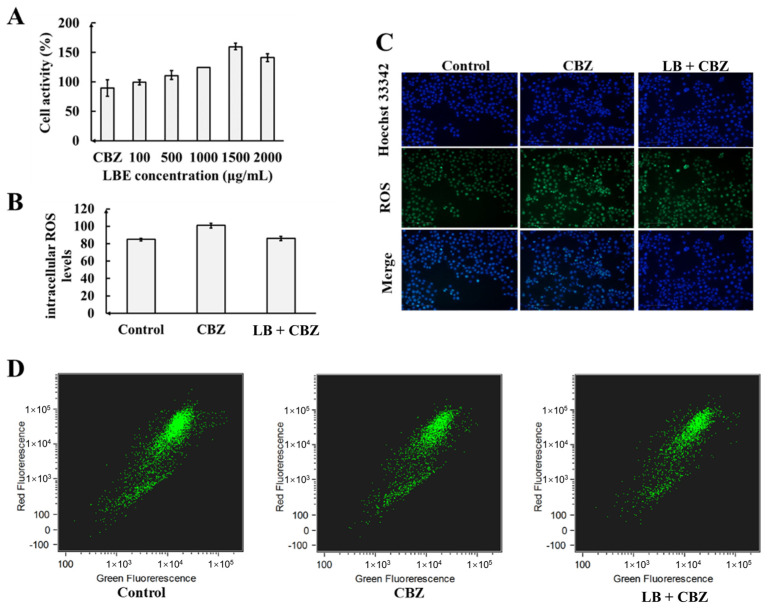
Effects of different concentrations of LBE on activity of PC12-damaged cells (**A**), ROS levels (**B**), and high-content screening images of PC12 cells after LBE pretreatment for 2 h and 24 h exposure to CBZ (**C**); effects of LB on CBZ-induced MMP changes using JC-1 (**D**).

The sample obtained at 100 W of ultrasonication at 30 °C for 15 min was diluted to 100, 500, 1000, 1500, and 2000 μg/mL with complete medium. PC12 cells were pretreated with the LBE solution for 2 h, followed by the addition of 15.9 μmol/L CBZ for 24 h. The results of the CCK-8 assays (Figure 3A) showed that the cell viability in the CBZ-treated group decreased to 90% compared with that in the control group. Furthermore, the application of the LBE solution at concentrations between 100 and 2000 μg/mL led to a marked increase in cell viability, compared with the CBZ-treated cells. Furthermore, 1500 μg/mL of LBE solution was found to exhibit the most significant protection against the effects of 15.9 μmol/L CBZ, resulting in cell viability of up to 159%. Therefore, 1500 μg/mL LBE solution was chosen for the subsequent experiments.

According to the literature, the content of AA-2βG in LB fruit is 0.5–1.2% [6,23]. The findings showed that 60 μmol/L (20.296 mg/L) of AA-2βG was the most effective, providing 140% protection against CBZ-induced damage to PC12 cells. Therefore, the optimal concentration of AA-2βG, based on the conversion of the most effective LBE concentration, was 7.5–18 mg/L, consistent with the results of the AA-2βG standard product. The content of CGA in LB fruit is 0.007–0.02% [24,25]. The verification process for CGA was the same as that used for AA-2βG, showing that 60 μmol/L (21.258 mg/L) of CGA was the most effective, providing 162% protection against CBZ-induced damage to PC12 cells. The optimal concentration of CGA, based on the conversion of the most effective LBE concentration, was 1.05–3 mg/L, consistent with the results of the CGA standard product.

### 3.4. Analysis of the Antioxidant Effects of LBE on PC12 Cells

#### 3.4.1. Effect of LBE on CBZ-Induced Changes in ROS Levels in PC12 Cells

The high-content imaging results are presented in Figure 3C. The blue fluorescence represents the total DNA content within the cells, primarily used for cell localization and quantification. The CellROX^®^ Green reagent emits bright green fluorescence in the presence of intracellular ROS, with stronger intensities indicating higher levels of intracellular ROS. The bar chart in Figure 3 shows the amount of ROS per cell after the different treatments. As shown in Figure 3B, CBZ-treated PC12 cells showed markedly increased ROS levels (18.6% higher) compared with the control group. Conversely, compared with the CBZ group, the pretreatment of PC12 cells with LBE resulted in 14.6% reductions in ROS, effectively inhibiting ROS production.

#### 3.4.2. Effect of LBE on CBZ-Induced MMP Changes in PC12 Cells

The primary origin of ROS in cells is attributed to the mitochondria. The dysfunction of these organelles can disrupt the intracellular calcium homeostasis, leading to ROS accumulation and intensifying the cellular response to oxidative stress [26]. Notably, the mitochondrial electron transport chain (ETC) system represents the major contributor to intracellular ROS generation [27]. Previous studies have demonstrated the importance of the MMP in the assessment of mitochondrial function. As the MMP typically declines when mitochondrial function is damaged, absent or reduced MMP is one of the indicators of mitochondrial dysfunction [28]. The current findings demonstrated that LBE effectively increased the MMP in PC12 cells after treatment with CBZ, while concurrently suppressing ROS production.

Figure 3D shows the results of the examination of PC12 cells with the JC-1 reagent. The vertical axis represents the intensity of the red fluorescence, while that of the green fluorescence is shown on the horizontal axis. The calculation of the ratio between the red and green fluorescence showed that it was significantly reduced by 12.4% in the CBZ-induced PC12 cells, while the ratio was markedly increased by 12.0% in the LBE group compared with the CBZ group, showing that LBP treatment could effectively reverse the MMP decline.

The findings demonstrate that LBE increased the MMP in CBZ-treated PC12 cells, while concurrently suppressing ROS production. It can be inferred that LBE facilitates the restoration of mitochondrial function through its inhibitory effect on excessive ROS generation triggered by CBZ.

#### 3.4.3. Effect of LBE on CBZ-Induced Gene Expression Changes in PC12 Cells and Enrichment Analysis

**Figure 4:**
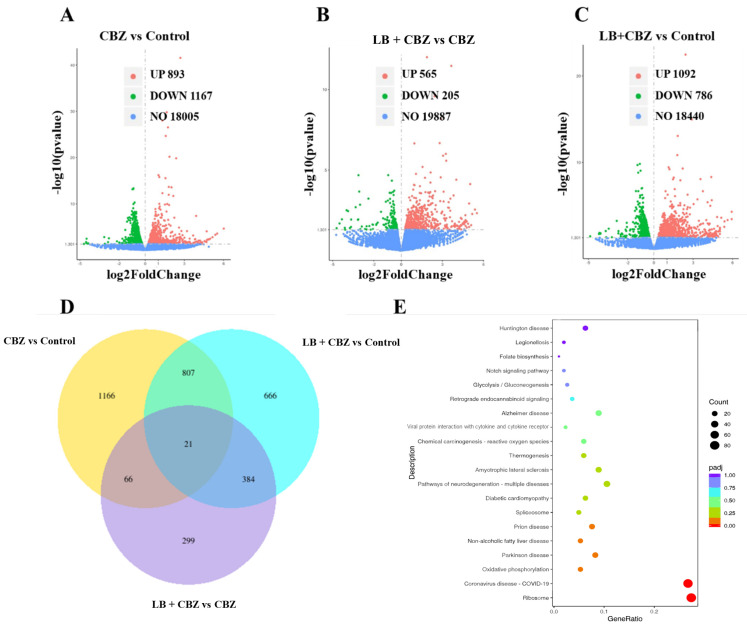
Volcano plot of differential genes for CBZ vs. control (**A**), LB + CBZ vs. CBZ (**B**), and LB + CBZ vs. control (**C**); Venn diagram of differential genes across comparison groups (**D**); LB + CBZ vs. CBZ group KEGG enrichment analysis scatter plot (**E**).

Differentially expressed genes among the control, CBZ, and LB + CBZ groups were identified using the criteria of *p* ≤ 0.05 and |log2FoldChange| ≥ 0. Volcano plots of the CBZ vs. control, LB + CBZ vs. CBZ, and LB + CBZ vs. control groups were plotted with the log2FoldChange as the X-axis and −log10*p*value as the Y-axis. As shown in Figure 4A–C, 2060, 1878, and 770 differentially expressed genes were identified in the CBZ vs. control, LB + CBZ vs. control, and LB + CBZ vs. CBZ groups, respectively. It should be noted that the number of differentially expressed genes was lower in the LB + CBZ vs. control group compared with the CBZ vs. control group, indicating that the pretreatment of PC12 cells with LB could reduce many of the effects of CBZ on gene expression. Furthermore, unique or common differentially expressed genes associated with the three groups were identified using Venn diagrams. As shown in Figure 3D, 21 differentially expressed genes, namely Abcb1b, Mrps16, Ypel3, Tcp11l2, Rps28, ENSRNOG00000064142, ENSRNOG00000067986, ENSRNOG00000062337, Lcn3, ENSRNOG00000065988, Rps4x, Rpl32-ps3, Rps15al4, novel.1021, Muc15, Rps19, RGD1564606, Matk, novel.233, ENSRNOG00000069617, and Fosb, were found to be common among the three groups, suggesting the association of these genes with the antioxidant effects of LB on CBZ-damaged PC12 cells.

A total of 3409 differentially expressed genes were identified between the different transcriptomic samples. This large number of genes complicated the analysis. Therefore, the genes were classified according to their enrichment in specific pathways, allowing an analysis of the molecular mechanism underlying the antioxidative effects of fresh LBE on CBZ-damaged PC12 cells. The Gene Ontology (GO) database provides detailed annotations of gene functions, divided into three categories, namely biological processes (BP), cellular components (CC), and molecular functions (MF). In the present study, the GO functional enrichment analysis of the 3409 differentially expressed genes was conducted using the clusterProfiler software (v 4.12.1). The threshold for significant enrichment was set at *p* < 0.05. As shown in Figure S1, compared to the control group, the differentially expressed genes in the CBZ group were found to be significantly enriched in biological processes such as chromosome segregation, nuclear division, and the regulation of the mitotic cell cycle. Most of these differentially expressed genes are located in the centromeric regions of chromosomes and interact catalytically with DNA. Furthermore, the enrichment of genes in the LB + CBZ group vs. CBZ was mainly associated with biological processes such as cytoplasmic translation and ribosomal small subunit assembly. These genes were mostly associated with ribosomes, where they were involved in the MF category of ribosome formation and rRNA binding. These results indicate that cell division-related biological processes, such as chromosome segregation and nuclear division, were significantly affected when PC12 cells experienced oxidative stress caused by CBZ. Treatment with LBE led to significant changes in the GO BP and MF categories associated with ribosomes.

A KEGG enrichment analysis was performed to investigate the antioxidant effects of fresh LBE, using a significance threshold of *p* < 0.05. The comparison between the LBE + CBZ and CBZ groups is illustrated in Figure 4E. As shown in the figure, enrichment was observed in pathways associated with ribosomes and OXPHOS, indicating the importance of these pathways in the protective mechanism. Ribosomes are known to be involved in protein synthesis and are essential for cell growth, development, and survival.

Genes related to protein processing and protein export pathways in ribosomes were significantly enriched and upregulated after LBE treatment. This may represent a compensatory response to resist the adverse effects of oxidative stress and apoptosis, which is in line with the results obtained using GO. Meydan et al. [29] indicated that oxidative stress causes ribosomes to pause at certain amino acid motifs. Mitochondria, both the sources and targets of ROS, are highly dynamic organelles that have a crucial role in maintaining the cellular balance by regulating the ATP levels and generating minimal amounts of ROS for cellular communication [30]. OXPHOS occurs mainly in the mitochondria, and the inhibition of OXPHOS increases mitochondrial ROS production. Li et al. [31] examined the effects of 1-NP on mitochondrial function and ROS in Leydig cells. Reductions in MMP, OXPHOS, and ATP were found to be closely associated with increased ROS production. The mitochondrial electron transport chain is a redox center that regulates internal homeostasis by generating ATP and producing ROS [32]. The KEGG enrichment analysis of the transcriptomic data showed the upregulation of the OXPHOS pathway, while the MMP evaluations (Figure 3D) also indicated that it returned to normal after LBE treatment. Five OXPHOS subunits, ATP5m, Uqcrq, Cox7c, Ndufa, and Mtnd3, were upregulated. Cox7c, a protein associated with cytochrome c, is a catalyst that facilitates electron transfer from reduced cytochrome c to oxygen [33]. The upregulation of these genes led to the restoration of both the ETC and MMP.

#### 3.4.4. Effects of LBE on CBZ-Induced Metabolites Changes in PC12 Cells and Pathway Analysis

**Figure 5:**
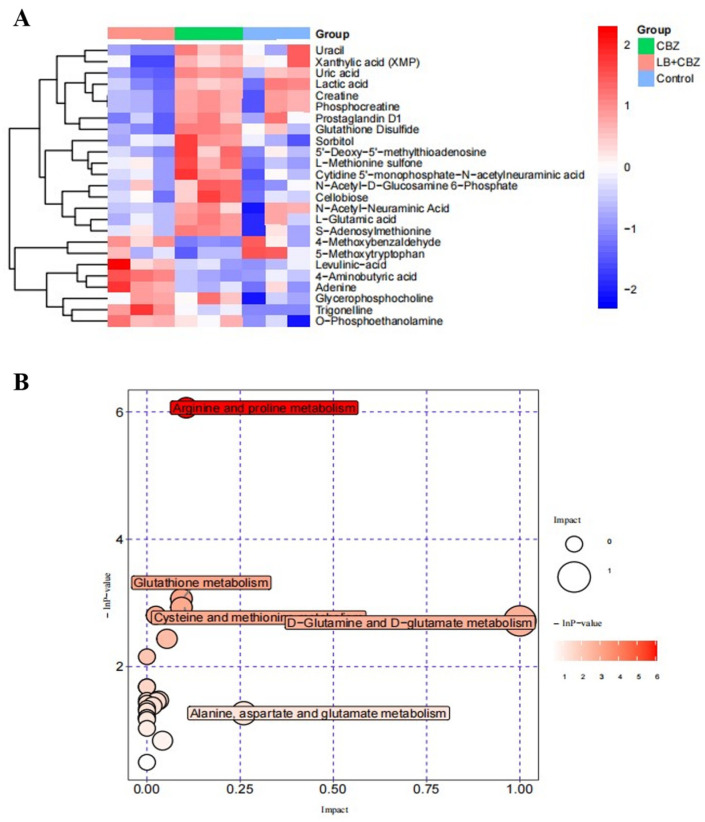
Heatmap of hierarchical clustering analysis of 25 differential metabolites (**A**) and metabolic pathway analysis for LB + CBZ vs. CBZ vs. control group (**B**). Note: the size and color of the circle represent the pathway effect and pathway significance, respectively.

The calibration curves and R^2^ values of 251 compounds are shown in Table S2, and good linearity was obtained with R^2^ ≥ 0.99 for all analytes. A total of 237 metabolites remained after removal and de-noising using the relative standard deviation. The missing values were then substituted with half of the minimum value. The analysis of the differential abundance of metabolites between the LB + CBZ, CBZ, and control groups identified 25 metabolites (*p* < 0.05). The heatmap of these 25 metabolites is shown in Figure 5A, and detailed information on the metabolites is provided in Table S3. Specifically, the abundance of most metabolites, including lactic acid, uracil, levulinic acid, creatine, phosphocreatine, and adenine, was increased in the CBZ-induced group compared with the control group, with only three metabolites showing reduced abundance, namely 4-aminobutyric acid, 4-methoxybenzaldehyde, and 5-methoxytryptophan. It should be noted that the abundance of these three metabolites was increased in the LB + CBZ group compared with the CBZ group, while that of most other metabolites was reduced. These results indicate that LB treatment could restore the changes in metabolites caused by CBZ to some extent.

Generally, metabolism is regulated by complex pathways and networks of genes and proteins. Therefore, the metabolic pathways associated with 25 identified differentially expressed genes were investigated using the KEGG database. The key pathways most significantly associated with different treatments were identified by enrichment and topological analyses, and the results are shown in the bubble diagram in Figure 5B. It can be seen that metabolites showing differential abundance between the LB + CBZ, CBZ, and control groups were mainly enriched in several metabolic pathways, including arginine and proline metabolism, D-glutamine and D-glutamate metabolism, cysteine and methionine metabolism, glutathione (GSH) metabolism, and alanine, aspartate, and glutamate metabolism.

L-arginine is generated by nitric oxide synthase (NOS) and is the direct precursor of nitric oxide (NO). This molecule acts as a widespread signaling factor and plays an important role in various cellular functions [34]. Zhang et al. [35] reported that supplementation with L-arginine could improve the balance between ROS production and antioxidant defense by scavenging the excess ROS induced by LPS. The scavenging effect of arginine on ROS was also demonstrated in a study conducted by Mei et al. [36]. However, excessive levels of arginine may cause damage to the CNS, as the arginine levels have been found to be significantly increased in the cerebrospinal fluid of patients with Alzheimer’s disease compared with individuals with mild cognitive impairment [37]. Ornithine is produced during arginine metabolism, and glutamate can be converted into proline by the arginine and proline metabolic pathways. Proline, the only secondary amino acid found in proteins, provides physical stability, serving as the foundation for molecular recognition and signal transduction. Mayneris-Perxachs et al. [38] indicated that disturbances in proline metabolism affect the balance between the glutamatergic and GABAergic systems. Gao et al. [39] also suggested that baicalein could protect PC12 cells against Aβ25-35-induced cytotoxicity, which may be associated with arginine and proline metabolism. Overall, the arginine and proline metabolic pathways can act as both scavengers and producers of ROS. Thus, the maintenance of arginine and proline homeostasis is crucial for cellular function.

Glutamate, the precursor of arginine biosynthesis, is the main excitatory neurotransmitter [40]. The dysregulation of glutamate may lead to neuronal apoptosis, which has been found to be associated with chronic neurodegenerative diseases [41]. The downregulation of glutamate and upregulation of arginine were observed in PC12 cells in a study of the mechanism underlying the cytotoxicity of BDE-47 [42]. Similarly, corticosterone has been shown to induce neurotoxicity in PC12 cells through the downregulation of glutamate, leading to disordered D-glutamine and D-glutamate metabolism, as well as GSH metabolism, while liquiritin-mediated metabolic regulation may protect against corticosterone-induced neurotoxicity in PC12 cells [43]. GSH is composed of three amino acids, namely L-cysteine, L-glutamine, and glycine. The primary mechanism used by cells to maintain ROS homeostasis and reduce oxidative stress is the oxidation of GSH to its disulfide dimer GSSG [44]. Long et al. [45] observed that deoxynivalenol induced oxidative stress by increasing the GSSG levels in IPEC-J2 cells. In our study, we also observed increased GSSG levels after CBZ induction. However, this was reversed by the treatment with LBE.

#### 3.4.5. Integrated Analysis of the Antioxidative Mechanism of LBE on CBZ-Damaged PC12 Cells

**Figure 6:**
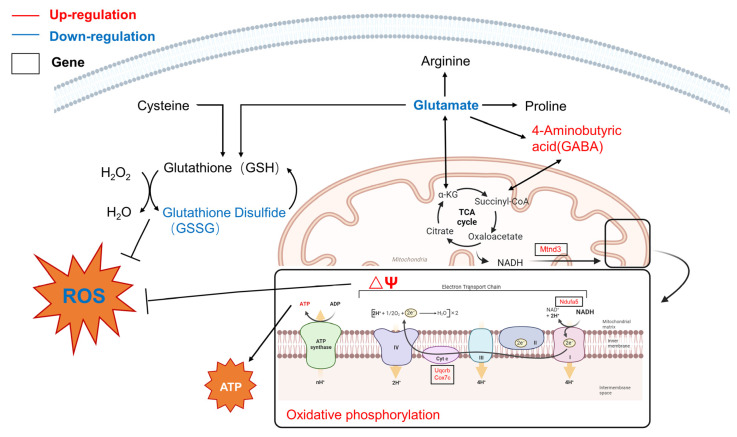
Schematic diagram of the antioxidative mechanism of LBE in CBZ-damaged PC12 cells.

Combined with the results of the ROS levels and MMP changes in PC12 cells, as well as transcriptomics and targeted metabolomics, it was concluded that the effects of LBE were associated with mitochondrial homeostasis, ATP production, and the GSH/peroxidase oxygen-reactive protein system (Figure 6). The pathways of D-glutamine and D-glutamate metabolism; cysteine and methionine metabolism; GSH metabolism; and alanine, aspartate, and glutamate metabolism were significantly enriched following pretreatment with LBE. This was related to the significant observed changes in the expression of ribosomal-related genes and the GO and KEGG results described in Section 3.4.3. Among these amino acids, cysteine and glutamate are involved in the GSH cycle for the removal of excess ROS in the cell. Moreover, other amino acids, such as glutamate, 4-aminobutyric acid (GABA), and aspartic acid, participate in the TCA cycle to produce substrates for OXPHOS in the ETC. After LBE treatment, the upregulation of OXPHOS subunits such as Uqcrq, Cox7c, and Ndufa indicated the reversal of ETC inhibition and restoration of the MMP, thus preventing the generation of excessive ROS caused by mitochondrial damage. In summary, LBE maintains redox homeostasis by (i) activating OXPHOS and restoring the MMP to prevent mitochondrial damage; (ii) maintaining the stability of the TCA cycle through the regulation of amino acid metabolic disorder to generate ATP; and (iii) regulating the GSH metabolic pathway to remove excess ROS in cells.

## 4. Conclusions

In conclusion, the CBZ-induced PC12 cell injury model was constructed successfully using a CBZ concentration of 15.9 μmol/L. Moreover, ultrasonication at 100 W for 15 min at a temperature of 30 °C was found to be optimal for LBE extraction, as shown by the results of the FRAP and DPPH radical scavenging assays, and an LBE concentration of 1500 μg/mL was found to be the most effective for the protection of PC12 cells from injury induced by CBZ. These conditions were selected for the investigation of the antioxidant mechanism of LBE. The pretreatment of PC12 cells with LBE resulted in a 14.6% reduction in ROS, effectively inhibiting ROS production, which facilitated the restoration of mitochondrial function mediated by its inhibition of ROS generation triggered by CBZ. Combined with the analysis of the changes in ROS and the MMP in PC12 cells, transcriptomics, and metabolomics, it was concluded that LBE was involved in mitochondrial homeostasis, ATP production, and the glutathione/peroxidase oxygen-reactive protein system, and that LBE maintains redox homeostasis mainly by activating OXPHOS and restoring the MMP, thus maintaining the stability of the TCA cycle and regulating the glutathione metabolic pathway to promote the removal of excess ROS. However, this study also had some limitations. Firstly, the detailed composition of LBE, especially in terms of its functional components, could be further investigated. The effects of specific functional components on cells and how they interact with each other could also be further studied. In addition, the dose-effect relationship could be investigated using animal experiments or other techniques. Overall, this study contributes further insights for the understanding of the mechanism underlying the antioxidant activity of fresh LBE. The results also have important implications for the risk–benefit analysis of LB consumption.

## Data Availability

The original contributions presented in the study are included in the article/supplementary material, further inquiries can be directed to the corresponding authors.

## Supplementary Materials

The following supporting information can be downloaded at: https://www.mdpi.com/article/10.3390/foods13152384/s1. Table S1 DOE trial design for significant impact factors. Table S2 The standard curve of 251 compounds. Table S3 The information of 25 differential metabolites. Figure S1 The standard curves for total phenols (A) and flavonoids (B). Figure S2 GO enrichment analysis histogram for each comparison groups (CBZ vs Control (A), LB + CBZ vs. CBZ (B)).

## References

[1] Skenderidis P., Lampakis D., Giavasis I., Leontopoulos S., Petrotos K., Hadjichristodoulou C., Tsakalof A. (2019). Chemical properties, fatty-acid composition, and antioxidant activity of Goji berry (*Lycium barbarum* L. and *Lycium chinense* Mill.) Fruits. Antioxidants.

[2] Zhang H., Liu F., Wang J., Yang Q., Wang P., Zhao H., Wang J., Wang C., Xu X. (2021). Salicylic acid inhibits the postharvest decay of goji berry (*Lycium barbarum* L.) by modulating the antioxidant system and phenylpropanoid metabolites. Postharvest Biol. Technol..

[3] Wang Y., Liang X., Guo S., Li Y., Zhang B., Yue Y., Wei A., Cao Y., Zhao J. (2019). Evaluation of nutrients and related environmental factors for wolfberry (*Lycium barbarum*) fruits grown in the different areas of China. Biochem. Syst. Ecol..

[4] Yang J., Wei Y., Ding J., Li Y., Ma J., Liu J. (2018). Research and application of *Lycii Fructus* in medicinal field. Chin. Herb. Med..

[5] Roy U., Cathirose P. (2020). Lycium (goji) Berry Standards of Identity Analysis, Quality Control, and Therapeutics.

[6] Toyoda-Ono Y., Maeda M., Nakao M., Yoshimura M., Sugiura-Tomimori N., Fukami H. (2004). 2-O-(beta-D-Glucopyranosyl)ascorbic acid, a novel ascorbic acid analogue isolated from *Lycium* fruit. J. Agric. Food Chem..

[7] Zhang H., Ma Z., Wang J., Wang P., Lu D., Deng S., Lei H., Gao Y., Tao Y. (2021). Treatment with exogenous salicylic acid maintains quality, increases bioactive compounds, and enhances the antioxidant capacity of fresh goji (*Lycium barbarum* L.) fruit during storage. LWT-Food Sci. Technol..

[8] Xing L., Wang Y., Luo R., Li X., Zou L. (2021). Determination of 31 pesticide residues in wolfberry by LC-MS/MS and dietary risk assessment of wolfberry consumption. Food Sci. Technol..

[9] Fang Z., Zhang Y., Lü Y., Ma G., Chen J., Liu D., Ye X. (2009). Phenolic compounds and antioxidant capacities of bayberry juices. Food Chem..

[10] Leonard W., Xiong Y., Zhang P., Ying D., Fang Z. (2021). Enhanced lignanamide absorption and antioxidative effect of extruded hempseed (*Cannabis sativa* L.) hull in Caco-2 intestinal cell culture. J. Agric. Food Chem..

[11] Valko M., Leibfritz D., Moncol J., Cronin M.T., Mazur M., Telser J. (2007). Free radicals and antioxidants in normal physiological functions and human disease. Int. J. Biochem. Cell Biol..

[12] Vandereyken K., Sifrim A., Thienpont B., Voet T. (2023). Methods and applications for single-cell and spatial multi-omics. Nat. Rev. Genet..

[13] Mayneris-Perxachs J., Anna C., María A., Martin M., Lisset V., Burokas A., Blasco G., Coll C., Escrichs A., Biarnés C. (2020). Translational Genomics in Neurocritical Care: A Review. Neurotherapeutics.

[14] Lin C., Sang Q., Fu Z., Yang S., Zhang M., Zhang H., Wang Y., Hu P. (2023). Deciphering mechanism of Zhishi-Xiebai-Guizhi decoction against hypoxia/reoxygenation injury in cardiomyocytes by cell metabolomics: Regulation of oxidative stress and energy acquisition. J. Chromatogr. B.

[15] Gao Y., Zhu J., Sun M., Wang S., Liu H. (2022). Metabolomics study based on GC–MS reveals a protective function of luteolin against glutamate-induced PC12 cell injury. Biomed. Chromatogr..

[16] Bhadange Y.A., Saharan V.K., Sonawane S.H., Boczkaj G. (2022). Intensification of catechin extraction from the bark of Syzygium cumini using ultrasonication: Optimization, characterization, degradation analysis and kinetic studies. Chem. Eng. Process.-Process Intensif..

[17] Zhang D.Y., Wan Y., Xu J.Y., Wu G.H., Li L., Yao X.H. (2016). Ultrasound extraction of polysaccharides from mulberry leaves and their effect on enhancing antioxidant activity. Carbohydr. Polym..

[18] Shen Y., Zhang H., Cheng L., Wang L., Qian H., Qi X. (2016). In vitro and in vivo antioxidant activity of polyphenols extracted from black highland barley. Food Chem..

[19] Song P., Sun M., Liu C., Liu J., Lin P., Chen H., Zhou D., Tang K., Wang A., Jin Y. (2023). Reactive oxygen species damage bovine endometrial epithelial cells via the cytochrome C-mPTP pathway. Antioxidants.

[20] Tang W., Fan W., Wang Z., Zhang W., Zhou S., Liu Y., Yang Z., Shao E., Zhang G., Jacobson O. (2018). Acidity/reducibility dual-responsive hollow mesoporous organosilica nanoplatforms for tumor-specific self-assembly and synergistic therapy. ACS Nano.

[21] Skenderidis P., Petrotos K., Giavasis I., Hadjichristodoulou C., Tsakalof A. (2017). Optimization of ultrasound assisted extraction of of goji berry (*Lycium barbarum*) fruits and evaluation of extracts’ bioactivity. J. Food Process Eng..

[22] Ma R., Sun X., Yang C., Fan Y. (2023). Integrated transcriptome and metabolome provide insight into flavonoid variation in goji berries (*Lycium barbarum* L.) from different areas in China. Plant Physiol. Biochem..

[23] Zhu B., Zhang W., Qin Y., Zhao J., Li S. (2022). Quality evaluation of *Lycium barbarum* L. fruits from different regions in China based on 2-O-β-D-glucopyranosyl-L-ascorbic acid. J. Food Compos. Anal..

[24] Inbaraj B.S., Lu H., Kao T.H., Chen B.H. (2010). Simultaneous determination of phenolic acids and flavonoids in *Lycium barbarum* Linnaeus by HPLC–DAD–ESI-MS. J. Pharm. Biomed. Anal..

[25] Zhao W., Shi Y. (2022). Comprehensive analysis of phenolic compounds in four varieties of goji berries at different ripening stages by UPLC–MS/MS. J. Food Compos. Anal..

[26] Guo C., Sun L., Chen X. (2013). Oxidative stress, mitochondrial damage and neurodegenerative diseases. Neural Regen. Res..

[27] Wang D.D., Jin M.F., Zhao D.J., Ni H. (2019). Reduction of mitophagy-related oxidative stress and preservation of mitochondria function using melatonin therapy in an HT22 hippocampal neuronal cell model of glutamate-induced excitotoxicity. Front. Endocrinol..

[28] Zhang R., Niu G., Li X., Guo L., Zhang H., Yang R., Chen Y., Yu X., Tang B.Z. (2019). Reaction-free and MMP-independent fluorescent probes for long-term mitochondria visualization and tracking. Chem. Sci..

[29] Meydan S., Barros G.C., Simões V., Harley L., Cizubu B.K., Guydosh N.R., Silva G.M. (2023). The ubiquitin conjugase Rad6 mediates ribosome pausing during oxidative stress. Cell Rep..

[30] He Y., Yang Z., Pi J., Cai T., Xia Y., Cao X., Liu J. (2022). EGCG attenuates the neurotoxicity of methylglyoxal via regulating MAPK and the downstream signaling pathways and inhibiting advanced glycation end products formation. Food Chem..

[31] Li J., Gao L., Chen J., Zhang W.W., Zhang X.Y., Wang B., Zhang C., Wang Y., Huang Y.C., Wang H. (2022). Mitochondrial ROS-mediated ribosome stalling and GCN2 activation are partially involved in 1-nitropyrene-induced steroidogenic inhibition in testes. Environ. Int..

[32] Vercellino I., Sazanov L.A. (2021). The assembly, regulation and function of the mitochondrial respiratory chain. Nat. Rev. Mol. Cell Biol..

[33] Zhu Y., Ma R., Cheng W., Qin M., Guo W., Qi Y., Dai J. (2024). Sijunzi decoction ameliorates gastric precancerous lesions via regulating oxidative phosphorylation based on proteomics and metabolomics. J. Food Compos. Anal..

[34] McDonald C.R., Cahill L.S., Gamble J.L., Elphinstone R., Gazdzinski L.M., Zhong K.J.Y., Philson A.C., Madanitsa M., Kalilani-Phiri L., Mwapasa V. (2018). Malaria in pregnancy alters L-arginine bioavailability and placental vascular development. Sci. Transl. Med..

[35] Zhang H., Peng A., Yu Y., Guo S., Wang M., Wang H. (2019). l-Arginine Protects Ovine intestinal epithelial cells from lipopolysaccharide-induced spoptosis through alleviating oxidative stress. J. Agric. Food Chem..

[36] Mei S., Chen X. (2023). Investigation into the anti-inflammatory mechanism of coffee leaf extract in LPS-induced Caco-2/U937 co-culture model through cytokines and NMR-based untargeted metabolomics analyses. Food Chem..

[37] Kaiser E., Schoenknecht P., Kassner S., Hildebrandt W., Kinscherf R., Schroeder J. (2010). Cerebrospinal fluid concentrations of functionally important amino acids and metabolic compounds in patients with mild cognitive impairment and Alzheimer’s disease. Neurodegener Dis..

[38] Mayneris-Perxachs J., Castells-Nobau A., Arnoriaga-Rodríguez M., Martin M., Vega-Correa L., Zapata C., Burokas A., Blasco G., Coll C., Escrichs A. (2022). Microbiota alterations in proline metabolism impact depression. Cell Metab..

[39] Gao L., Zhou F., Wang K.X., Zhou Y.Z., Du G.H., Qin X.M. (2020). Baicalein protects PC12 cells from Aβ(25)(-)(35)-induced cytotoxicity via inhibition of apoptosis and metabolic disorders. Life Sci..

[40] Tang Z., Li Y., Jiang Y., Cheng J., Xu S., Zhang J. (2019). Cellular metabolomics reveals glutamate and pyrimidine metabolism pathway alterations induced by BDE-47 in human neuroblastoma SK-N-SH cells. Ecotoxicol. Environ. Saf..

[41] Miladinovic T., Nashed M.G., Singh G. (2015). Overview of glutamatergic dysregulation in central pathologies. Biomolecules.

[42] He H., Shi X., Lawrence A., Hrovat J., Turner C., Cui J.Y., Gu H. (2020). 2,2′,4,4′-tetrabromodiphenyl ether (BDE-47) induces wide metabolic changes including attenuated mitochondrial function and enhanced glycolysis in PC12 cells. Ecotoxicol. Environ. Saf..

[43] Li X., Qin X., Tian J., Gao X., Wu X., Du G., Zhou Y. (1118). Liquiritin protects PC12 cells from corticosterone-induced neurotoxicity via regulation of metabolic disorders, attenuation ERK1/2-NF-κB pathway, activation Nrf2-Keap1 pathway, and inhibition mitochondrial apoptosis pathway. Ecotoxicol. Environ. Saf..

[44] Igbokwe C., Feng Y., Louis H., Benjamin I., Quaisie J., Duan Y., Tuly J.A., Cai M., Zhang H. (2024). Novel antioxidant peptides identified from coix seed by molecular docking, quantum chemical calculations and invitro study in HepG2 cells. Food Chem..

[45] Long H., Xin Z., Zhang F., Zhai Z., Ni X., Chen J., Yang K., Liao P., Zhang L., Xiao Z. (2021). The cytoprotective effects of dihydromyricetin and associated metabolic pathway changes on deoxynivalenol treated IPEC-J2 cells. Food Chem..

