# Supplementary material for: Investigation into Antioxidant Mechanism of Lycium barbarum Extract in Carbendazim-Induced PC12 Cell Injury Model through Transcriptomics and Metabolomics Analyses
Source: Foods. 2024 Jul 28;13(15):2384. doi: 10.3390/foods13152384 (PMC11311554; doi:10.3390/foods13152384)
Supplement: Supplementary file 1 [file foods-13-02384-s001.zip › foods-3051366-supplementary.pdf]

Table S1 DOE trial design for significant impact factors

Table S3 The information of 25 differential metabolites

Fig.S1 The standard curves for total phenols (A) and flavonoids(B).

Fig.S2 GO Enrichment Analysis Histogram for each comparison groups（CBZ vs Control（A）、LB+CBZ vs CBZ（B））

Table S1 DOE trial design for significant impact factors

Number	t	T	E
1	15	30	60
2	30	30	60
3	15	50	60
4	30	50	60
5	15	30	100
6	30	30	100
7	15	50	100
8	30	50	100

Table S3 The information of 25 differential metabolites

No	Metabolites	HMDB	MEAN Control	MEAN CBZ	MEAN LB+CBZ	NOVA P-VALUE
1	Lactic acid	HMDB0000190	251.5471	267.7306	176.3280	0.0445
2	4-Aminobutyric acid	HMDB0000112	0.1854	0.1457	0.5258	0.0001
3	Uracil	HMDB0000300	0.0271	0.0398	0.0025	0.0398
4	Levulinic-acid	HMDB0000720	0.0039	0.0042	0.0137	0.0486

5	Creatine	HMDB0000064	2.5282	3.0656	1.9278	0.0113
6	Phosphocreatine	HMDB0001511	2.5358	3.0748	1.9333	0.0112
7	Adenine	HMDB0000034	0.0003	0.0004	0.0006	0.0189
8	4-Methoxybenzaldehyde	HMDB0029686	0.6577	0.3787	0.7559	0.0115
9	Trigonelline	HMDB0000875	0.0000	0.0012	0.0032	0.0081
10	O-Phosphoethanolamine	HMDB0000224	0.3947	0.5849	0.6546	0.0168
11	L-Glutamic acid	HMDB0000148	29.5166	42.2237	29.7354	0.0097
12	Uric acid	HMDB0000289	0.9919	1.2538	0.5194	0.0036
13	L-Methionine sulfone	HMDB0062174	0.0125	0.0274	0.0167	0.0035
14	Sorbitol	HMDB0000247	1.3870	4.8588	2.0068	0.0084
15	5-Methoxytryptophan	HMDB0002339	0.0509	0.0326	0.0402	0.0481
16	Glycerophosphocholine	HMDB0000086	0.4557	0.6929	0.6822	0.0419
17	5'-Deoxy-5'-methylthioadenosine	HMDB0001173	0.0158	0.0272	0.0147	0.0127
18	N-Acetyl-D-Glucosamine-6-Phosphate	HMDB0001062	0.1165	0.1696	0.1314	0.0337
19	N-Acetyl-Neuraminic Acid	HMDB0000230	5.7998	7.3815	5.3403	0.0435
20	Cellobiose	HMDB0000055	14.0430	19.3510	14.3767	0.0235
21	Prostaglandin D1	HMDB0005102	4.5436	5.6338	2.8812	0.0466
22	Xanthylic acid (XMP)	HMDB0001554	0.0530	0.0539	0.0126	0.0451

23	S-Adenosylmethionine	HMDB0001185	2.6975	5.0288	3.5071	0.0346
24	Glutathione Disulfide	HMDB0003337	0.1460	0.2255	0.0731	0.0017
25	Cytidine 5'-monophosphate-N-acetylneuraminic acid	HMDB0001176	0.0521	0.0774	0.0553	0.0187

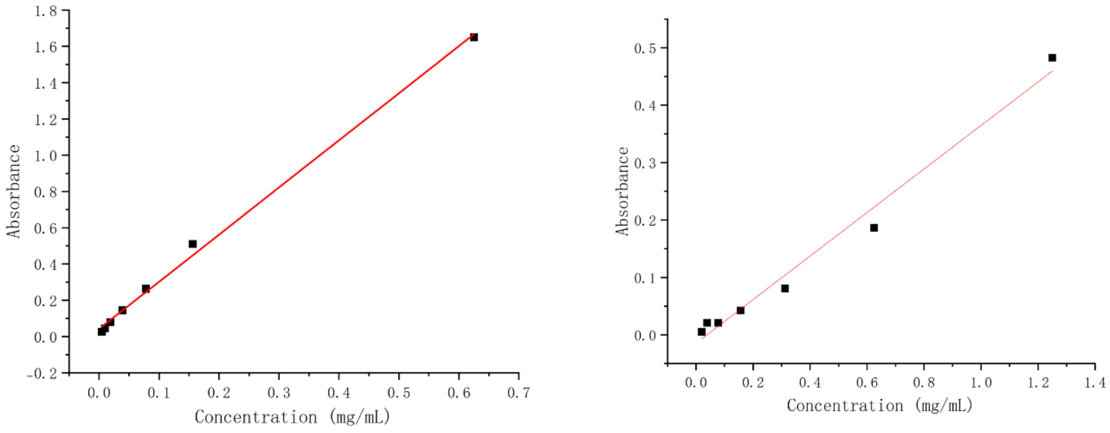

A **B**

Figure S1 The standard curves for total phenols (A) and flavonoids(B).

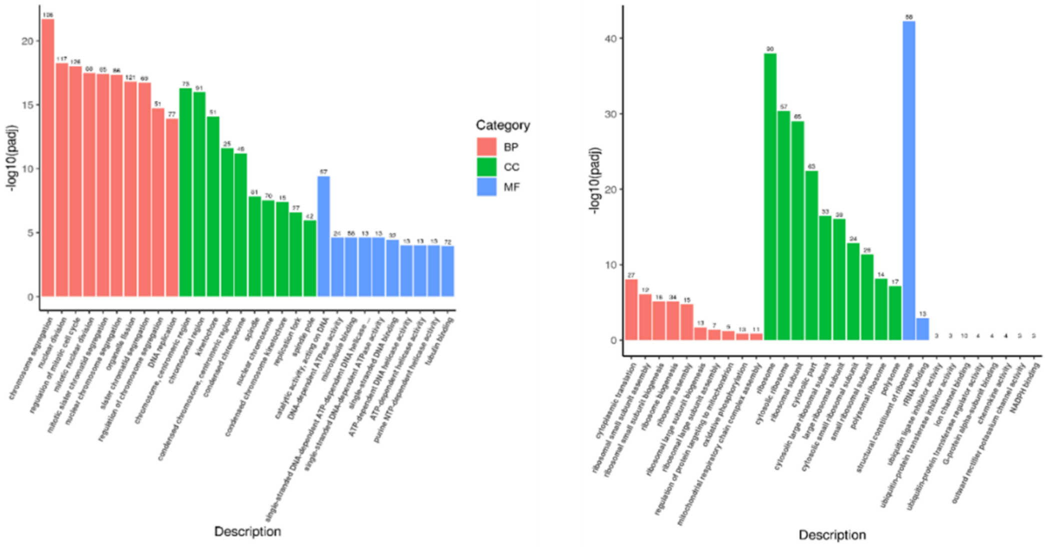

A **B**

Figure S2 eGO Enrichment Analysis Histogram for each comparison groups（CBZ vs Control（A）、LB+CBZ vs CBZ（B））
